# Comparative Review on the Aqueous Zinc-Ion Batteries (AZIBs) and Flexible Zinc-Ion Batteries (FZIBs)

**DOI:** 10.3390/nano12223997

**Published:** 2022-11-13

**Authors:** Md. Al-Amin, Saiful Islam, Sayed Ul Alam Shibly, Samia Iffat

**Affiliations:** 1 Department of Chemistry, University of Louisville, Louisville, KY 40292, USA; 2 Natural Science (Chemistry), American International University Bangladesh, Dhaka 1229, Bangladesh; 3 Basic Science Department, Primeasia University, Dhaka 1213, Bangladesh; 4 Telephone Shilpa Sangstha Ltd., Gazipur, Dhaka 1710, Bangladesh

**Keywords:** aqueous zinc-ion battery, flexible zinc-ion battery, anode and cathode materials

## Abstract

Lithium-ion batteries (LIBs) have been considered an easily accessible battery technology because of their low weight, cheapness, etc. Unfortunately, they have significant drawbacks, such as flammability and scarcity of lithium. Since the components of zinc-ion batteries are nonflammable, nontoxic, and cheap, AZIBs could be a suitable replacement for LIBs. In this article, the advantages and drawbacks of AZIBs over other energy storage devices are briefly discussed. This review focused on the cathode materials and electrolytes for AZIBs. In addition, we discussed the approaches to improve the electrochemical performance of zinc batteries. Here, we also discussed the polymer gel electrolytes and the electrodes for flexible zinc-ion batteries (FZIBs). Moreover, we have outlined the importance of temperature and additives in a flexible zinc-ion battery. Finally, we have discussed anode materials for both AZIBs and FZIBs. This review has summarized the advantages and disadvantages of AZIBs and FZIBs for future applications in commercial battery technology.

## 1. Introduction

Lithium-ion batteries (LIBs) have attracted much attention to battery technology due to their low weight, high energy densities, and specific power. Unfortunately, LIBs have major drawbacks, such as energy density limits, high cost, toxic nature, and safety issues [1,2]. Therefore, large-scale applications of LIBs are still challenging because of these limitations. However, lead-acid- and nickel-cadmium-based batteries are currently dominant in the present battery energy storage market due to their low cost and durability [1,3,4,5]. They have limitations such as poor energy densities and environmental problems due to toxic electrodes. Moreover, various alkali metal cations (such as Na^+^ and K^+^) and multivalent charge carriers (such as Mg^2+^, Al^3+^, and Zn^2+^) have been investigated in aqueous electrolyte-based batteries. For example, aqueous zinc-ion batteries are particularly appealing since Zn has a large natural abundance, a low redox potential, a high theoretical capacity, intrinsic safety, and low toxicity [1,2,5,6,7,8,9].

Aqueous zinc-ion batteries (AZIBs) are facing challenges due to the deteriorating effect of cathode and anode materials [10,11,12,13,14,15]. Hence, these effects are responsible for lowering the coulombic efficiency and the specific capacity of AZIBs. Scientists are still working to overcome these issues regarding AZIBs [1,2,3,5,6,7,8,15,16,17,18,19,20]. Additionally, they are giving more attention to modifying cathodes, which can enhance the electrochemical performance. Materials with a spinel or layer structure, such as Mn-based, vanadium-based, and Prussian blue analogous, are attracting attention for modifying electrode materials for AZIBs [11,21,22,23,24,25,26,27,28]. Among them, Mn-based materials have gained a lot of interest in the cathode materials because of their diverse crystal structures, different valence states, and high-voltage platforms [26,29,30,31,32]. First, alkaline electrolytes acting as primary electrolytes were used in Mn-based materials in the 1860s. Unfortunately, several challenges appeared in the alkaline electrolyte system in ZIBs, such as the dendrite and byproducts of the Zn anode resulting in low coulombic efficiency and poor cycle performance. In 1988, Yamamoto et al. used a mild acid ZnSO_4_ electrolyte in α-MnO_2_ to enhance the electrochemical performance, although this approach did not attract much scientific attention [33]. Similarly, Wang et al. have shown exceptional results when they used a moderate acid ZnSO_4_ electrolyte in α-MnO_2_, and it has shown good reversibility and cyclability [34]. However, aqueous zinc-ion batteries (AZIBs) are still under investigation, and the complexity arising from aqueous electrolytes is hindering the creation of highly cyclic stable ZIBs. Scientists are looking for suitable electrolytes in place of aqueous electrolytes to reduce these issues, such as dendrite formation, cathode dissolution, etc. They are considering that electrolytes that have a lesser water content might reduce the parasitic reaction and could improve cyclic stability as well as electrochemical performance.

**Figure 1:**
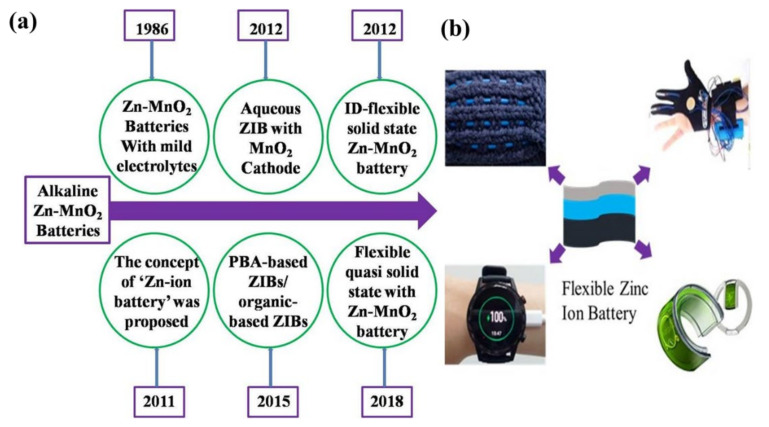
(**a**) A brief history of the development of ZIBs and FZIBs. (**b**) Exciting applications of flexible zinc-ion batteries.

Flexible zinc-ion batteries (FZIBs) are now being considered a sustainable selection to power portable electronics thanks to the development of aqueous rechargeable zinc-ion batteries (AZIBs) [35,36,37]. FZIBs with polymeric matrix electrolytes not only have better huge pile capabilities but also have better electrochemical behavior, as they eliminate dendrite formation and cathode dissolution [37,38,39,40,41,42]. As illustrated in Figure 1, flexible ZIBs have considerable potential for wearable applications due to their qualities such as exceptional flexibility, tolerance to deformation, and compatibility with the traditional textile industry. Several strategies for the fabrication of flexible ZIBs with both energy storage performance and mechanical robustness have been recently developed. However, the development of the strategy of ZIBs is still in its infancy. In this review, we provide recent updates on AZIBs and FZIBs.

## 2. Zinc-Ion Batteries (ZIBs)

**Figure 2:**
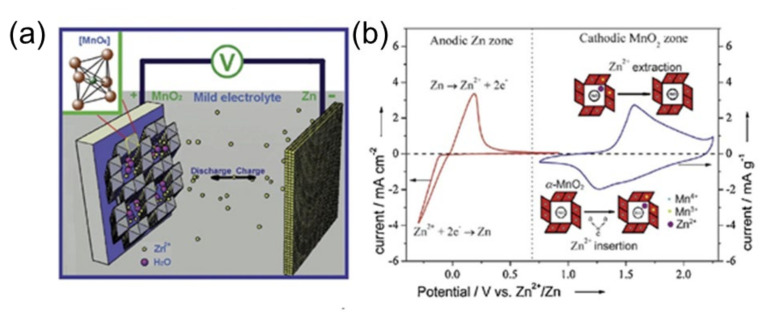
(**a**) A schematic illustration of aqueous ZIBs. (**b**) Zn storage mechanism of Zn/MnO_2_ system at anode and cathode sides. Reprinted with permission from Ref. [34]. Copyright 2011 Wiley.

Zinc-ion batteries consist of Zn metal as an anode and metal oxides as cathodes. Basically, Zn salt is used as an electrolyte for transporting Zn^2+^ ions during charge–discharge [34]. As illustrated in Figure 2a, MnO_2_ was used as a cathode, Zn foil was used as an anode, and mild aqueous ZnSO_4_ solution was utilized as an electrolyte. The reaction mechanism of Zn/MnO_2_ is as follows (Figure 2b):

Anode:

(1) $$\mathrm{Zn}\;↔{\mathrm{Zn}}^{2+}+2e^{-}$$

Cathode:

(2) $${\mathrm{Zn}}^{2+}+2e^{-}\;↔{\mathrm{ZnMn}}_{2}O_{4}$$

Here, Zn stripping and deposition happened at the anode and Zn insertion/extraction occurred at the cathode during the discharge–charge. Wei Sun et al. [43] and Buke Wu et al. [44] have proposed a new reaction mechanism and confirmed the Zn^2+^ and H+ co-insertion mechanism during discharge.

### 2.1. Advantages of ZIBs over Other Energy Storage Devices

**Figure 3:**
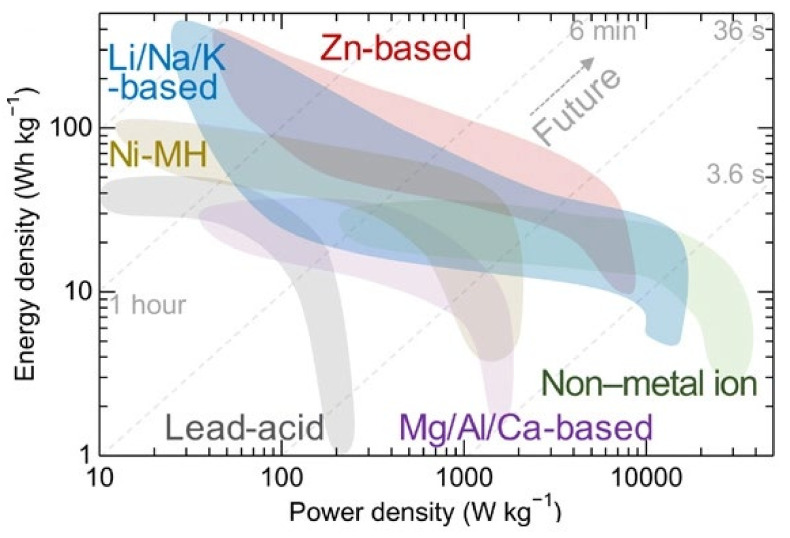
A comparative energy density curve for ZIBs and other energy storage systems. Reprinted with the permission from Ref. [45]. Copyright 2022 Science.

ZIBs have attracted much attention because of their lower cost, high storage capacity, etc. In addition, ZIBs are less flammable than lithium-ion batteries (LIBs). Although sodium-ion and potassium-ion are cheaper than LIBs, they also have flammability issues, like LIBs [1,2,3,4,5]. Above all, ZIBs are a suitable candidate for future-generation battery technology. Unfortunately, several barriers exist in the way of further implementations involving side products (hydrogen production, dissolution, and contact resistance) and zinc dendrite growth at the anode. These issues are hindering the creation of sustainable ZIBs. Hence, developing strategies of anodes and electrolytes and the reaction mechanisms are crucial. These developments could reduce cathode erosion, zinc dendrites’ formation, hydrogen evolution, and other issues [7,8,9,10]. It is assumed from the literature review of ZIBs that electrolytes and durable cathodes and anodic materials could play a pivotal role in making sustainable ZIBs. As a result, considerably more attention should be directed to electrolyte development technologies to produce greatly extended durability and consistency. As illustrated in Figure 3, the possibility of ZIB over other energy storage systems is because of the high energy density of the Zn batteries. In this section of the review, we will provide a more recent update on ZIBs. 

### 2.2. Zinc-Ion Batteries (ZIBs)—The Electrolytes and the Anode Materials

**Figure 4:**
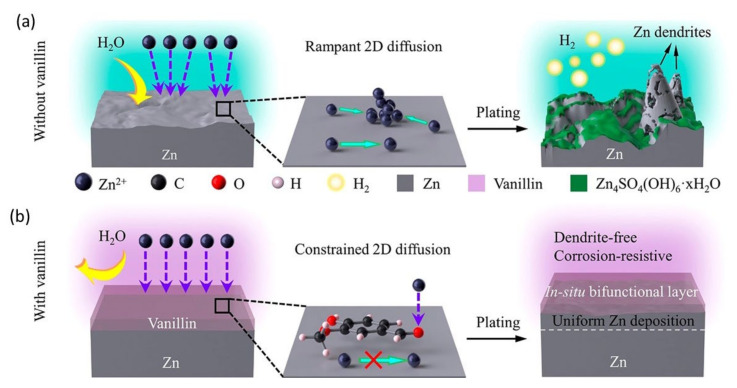
Schematics and bifunctional mechanism of vanillin sustaining Zn electrochemistry: Zn^2+^ diffusion/adsorption and Zn plating characteristics in (**a**) vanillin-free and (**b**) vanillin-containing aqueous ZnSO_4_ electrolytes are depicted schematically. Reprinted with permission from Ref. [55]. Copyright 2021 American Chemical Society.

Zinc anode has issues such as HER (hydrogen evolution reaction), dendrite formation, and shape abnormalities, which could play a significant role in degrading the cyclic stability and performance of ZIBs. These issues lead to a smaller coulombic efficiency. However, the cyclic stability and performance vary depending on the anode materials. Therefore, its performance is still poorer. One of the most popular and successful methods to mitigate adverse reactions and the growth of zinc dendrites is to add additives to electrolytes [11,18,24,25,26,29,30,46,47]. Two types of additives are generally employed in aqueous ZIBs, organic and inorganic. For example, organic additives, including polyethyleneimine (PEI), polyethylene glycol (PEG), poly(vinyl alcohol) (PVA), and diethyl ether, can bind on the tips of zinc patches to reduce local current density. Other organic additives such as benzotriazole (BTA), tetrabutylammonium bromide (TBAB), potassium persulfate (KPS), dimethyl sulfoxide (DMSO), benzotriazole (BTA), and ethanol can mostly adsorb on the anode surface, which can considerably reduce species diffusion and strong corrosion resistance. Additionally, some organic additives, such as ethylene glycol (EG), glucose, and DMSO, could impact the solvent evaporation process of solvated Zn^2+^ ions [42,48,49,50,51,52,53,54]. For example, the use of vanillin as a bifunctional additive in an aqueous electrolyte to stabilize Zn electrochemistry was reported by Cheng et al. [55]. The Zn anode, which was dipped in 5 mM of vanillin modified with a 2 M ZnSO_4_ electrolyte, has demonstrated the storage capacity (10 mAh cm^−2^ at 1 mA cm^−2^), cycling stability (1 mAh cm^−2^ for 1000 h), and coulombic efficiency (99.8%). In Figure 4, a clear effect of vanillin on Zn appeared.

**Figure 5:**
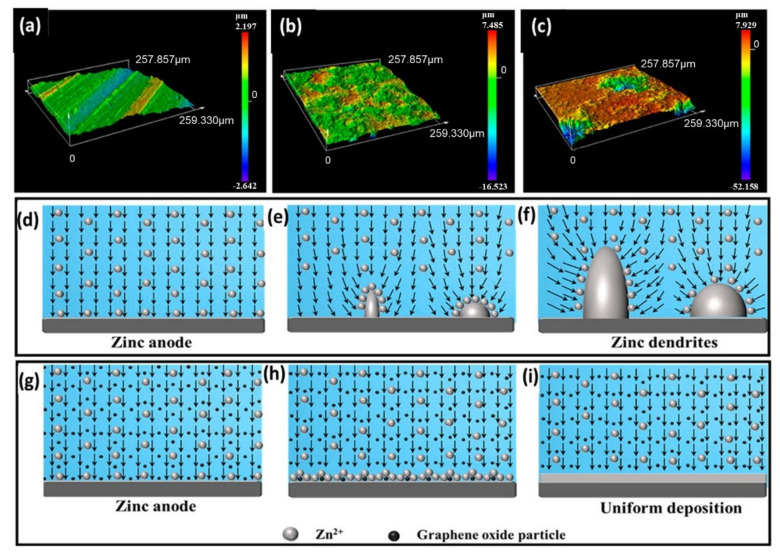
3D laser images of (**a**) the untouched zinc anode, (**b**) the zinc anode without GO additive, and (**c**) the zinc anode with GO additive after cycling. The electric field dispersal on the zinc anode (**d**–**f**) surface without GO electrolyte additive and (**g**–**i**) with GO electrolyte additive. The vectorial field describes the direction of the electric field. Reprinted with permission from Ref. [56]. Copyright 2021 American Chemical Society.

Without the vanillin additive in ZnSO_4_ electrolytes (Figure 4a), the apparent reaction between the aqueous solution and the thermodynamically unstable Zn anode incites parasitic reactions responsible for HER corrosion and a Zn_4_SO_4_(OH)_6_ xH_2_O byproduct. In addition, the horizontal movement of Zn^2+^ ions can invoke deposition on sporadically scattered prominent nuclei, which can limit surface energy and surface area, heterogeneous Zn plating/stripping, and promote dendritic development. The vanillin surface could not only cover active Zn from a free water attack through two-dimensional (2D) adsorption, but could also promote perpendicular Zn^2+^ migration and severely curb overgrown 2D diffusion, climaxing homogeneous and dendrite-free Zn building. Similarly, Qin et al. showed a hybrid electrolyte containing graphene oxide as a supplement that boosts consistent electric field distribution and eliminates Zn^2+^ nucleation over-potential, the smooth zinc electrodeposition layer, and reaction kinetics [56]. The symmetric zinc battery with graphene oxide addition consisted of a solid zinc anode that extended around 650 h at 1 mA cm^−2^ and kept a cycle life of 140 h at 10 mA cm^−2^. A 3D laser microscope was used to study the surface morphology of zinc anodes. In the initial condition, the electric field on the velvety surface of pure zinc foil was disseminated uniformly in the same order, as shown in Figure 5a–i.

**Figure 6:**
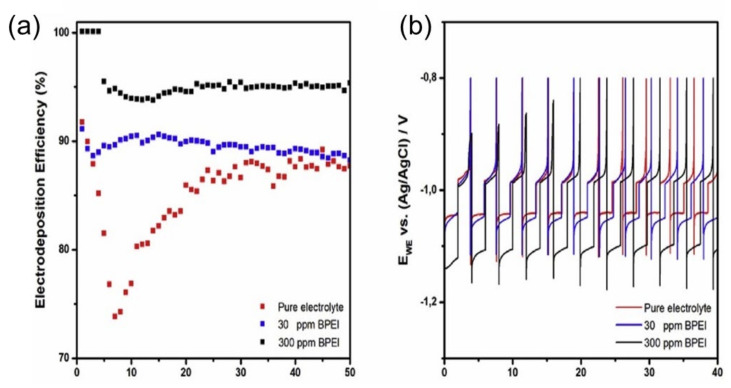
(**a**) Zinc electrodeposition efficiency upon 50 cycles without BPEI, and with 30 and 300 ppm of BPEI as an additive in 0.5 M of ZnSO_4_. (**b**) Potential variation of the working electrode during zinc deposition and dissolution in different solutions. Reprinted with permission from Ref. [53]. Copyright 2017 Elsevier.

When Zn^2+^ began to build up from a selected vicinity of the zinc floor, a distinguishable electric-powered subject turned established, and numerous outcomes confirmed that the neighborhood electric-powered subject after the dendrite tip turned into as much as two times that of a starting electrodeposit in the long run, mainly due to the formation of zinc ions within the dendrite tip (Figure 5e). Those indentations or dendrites popped up as purple regions within the 3D laser image (Figure 5b). Besides that, because the electrodeposit has become more potent and has been cut down the center into thinner dendrites (Figure 5f), the depth of the electrical subject ought to attain three instances or more than the preliminary electric-powered subject, indicating that the end effect enhanced until the battery demonstrated excessive impedance. Additionally, because of the inclination to lessen the floor power and decrease transmission paths, the presently exposed zinc coating has become the ultimate electrodeposition location. Using GO as an electrolyte addition, on the other hand, may completely repair the dendrite problem (Figure 5c). The bonding among GO debris in addition to Zn could not prevent distinguishable electric-powered fields from forming (Figure 5h) and could perhaps simply show the dissemination of the electrical subject; however, it may additionally confirm that charged Zn^2+^ operates right away to the anode floor (Figure 5i), resulting in a steady zinc deposition layer. Additionally, Mantia et al. tested the morphology and kinetics of zinc electrodeposition in a 0.5 M ZnSO_4_ solution with the use of branched polyethyleneimine (BPEI) as an electrolyte additive [53]. When BPEI is present, the electrodeposited layers undergo modifications from laminated hexagonal huge crystals to a compact layer, not using an essential boom form. Furthermore, it was discovered that deposition of BPEI at the substrate’s floor influences the kinetics of zinc electrodeposition and slows grain expansion, favoring nucleation overgrowth. As a result, BPEI guarantees that conductivity is calmly dispersed and that the deposited layer is uniform. Figure 6 illustrates the effectiveness of zinc electrodeposition with 30 and 300 ppm of BPEI, compared to the absence of BPEI. When no additive is introduced, the preliminary electrodeposition performance drops to much less than 75%, then steadily climbs to 88%.

The partial overlaying of zinc by zinc hydroxide or zinc oxide in the seventh cycle is noted to be the cause of this phenomenon. This prevents extra zinc from being electrodeposited, resulting in a vast preliminary over-potential that favors hydrogen formation. The minimum overall performance is observed when 30 ppm BPEI is added, whereby the overall performance is 88%. For the 4 primary cycles, the electrodeposition overall performance is 100% with the presence of 300 ppm BPEI. Following this, the performance is decreased to 95% and remains constant for the trial (Figure 6a). As a result, raising the additive from 30 to 300 ppm can increase the zinc electrodeposition overall performance from 88% to 95%. Furthermore, as proven in Figure 6b, BPEI adsorption does not affect the oxidation over-potential, however it does affect the cathodic over-potential, which might be 10 mV more with the presence of 30 ppm BPEI than with the natural electrolyte. The upward thrust in cathodic over-potential reaches 60 mV in the presence of 300 ppm BPEI.

### 2.3. Aqueous Zinc-Ion Batteries (AZIBs)—Cathode Materials

Sustainable cathodic materials of ZIBs require the following characteristics, such as a proper structure, excellent structural consistency, ideal working voltage, electrochemical stability, high energy storage density, and reduced cost and environmental consequences [1,2,3,4,5]. However, there are still several obstacles suppressing the advancement of this field. The most common issues are cathode dissolution byproducts. Scientists are trying to reduce these issues to make sustainable AZIBs. There are several cathode materials, such as manganese-based, vanadium-based, other materials-based, etc. Manganese-based material is a promising cathode material due to its lower cost, low toxicity, low combustible, high abundance, high storage capacity, and high ionic conductivity. Furthermore, the structural properties of MnO_2_ (α, β, γ, δ, λ) provide some adequate pathways and surfaces for the transport and accumulation of other metal ions, including Zn ions (Table 1). Chain-type, tunnel-type, and layered-type structures are interconnected to form MnO_6_ octahedra as the fundamental structural component by connecting vertices or edges [30,57,58,59,60]. These structural versatilities of MnO_2_ usually lead to making excellent cathode materials. 

The electrochemical execution shifted concurring with the different gem shapes. These tracts make the MnO_2_ reasonable for economical application in super-capacitors and lithium-ion batteries. For example, Yuan et al. showed that a manganese-based metal-organic system (MOF) can be utilized as a moved forward cathode for ZIBs [68]. The oxygen atoms of two neighboring -COO– are utilized to implement coordination unsaturation of Mn. Its moderately high reactivity and quick electrochemical response energy are upheld by its ideal unsaturated coordination degree, which offers prevalent Zn^2+^ transport and electron trade all through repetitive charging/discharging cycles. Due to the aforementioned characteristics, this MOF-based anode incorporates a huge capacity of 138 mAh g^−1^ at 100 mA g^−1^ and a long lifespan (93.5% capacity holding after 1000 cycles at 3000 mA g^−1^).

**Figure 7:**
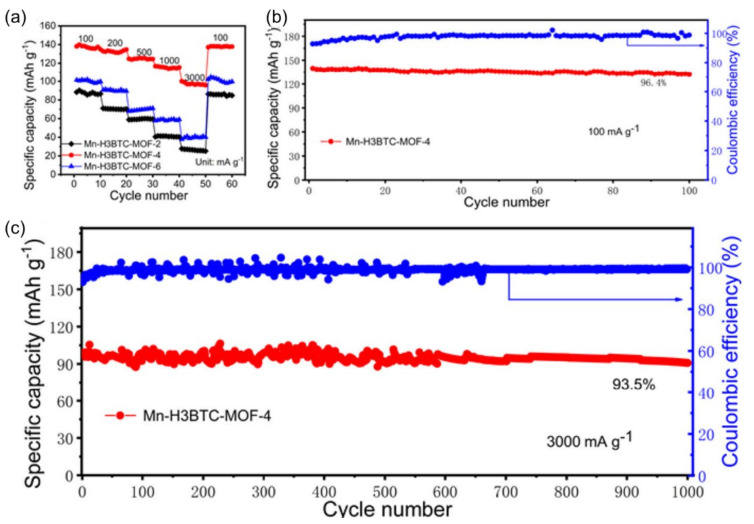
(**a**) The rate capability (from 100 to 3000 mA g^−1^) and (**b**,**c**) cycling performance of Mn-H_3_BTC-MOF-4 at 100 and at 3000 mA g^−1^, respectively. Reprinted with permission from Ref. [68]. Copyright 2021 American Chemical Society.

**Figure 8:**
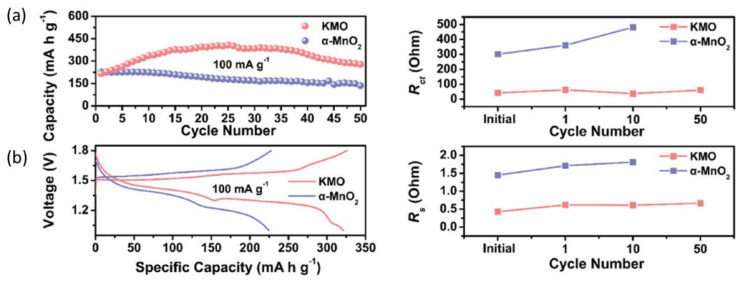
(**a**) Cycling performance at 100 mA g^−1^ and corresponding galvanostatic charge/discharge curves at the tenth cycle. (**b**) Plots of electrolyte resistance, Rs, and charge-transfer resistance, Rct. Reprinted with permission from Ref. [69]. Copyright 2019 Elsevier.

Figure 7a illustrates that as the current density increases from 100 to 3000 mA g^−1^, the distinctive volume of MnH_3_BTCMOF_4_ expands from 98 to 138 mAh g^−1^. This current density implies that it has higher capacity retention than MnH_3_BTCMOF_2_ and MnH_3_BTCMOF_6_. Cycle implementation of diverse cathode materials has been extensively studied (Figure 7b). The MnH_3_BTCMOF_4_ cathode shows remarkable specific volume retention of 96.4% after 100 cycles and 100% coulombic efficiency at 100 mA g^−1^. When built as a full battery, the device also shows a very long cycle stability with a Zn anode at 2 M Zn (CF_3_SO_3_), with 93.5% of the installed power after 1000 cycles at 3000 mA g^−1^ (Figure 7c). In addition, Liang et al. have reported that the potassium-ion stabilization and oxygen deficiency of K0.8Mn8O16 make it a high-energy and robust cathode for neutral aqueous ZIB [69]. They identified a strong energy output of 398 Wh kg^−1^ (depending on the mass of the cathode) and high stability over 1000 cycles, with no significant capacity loss. At 100 mA g^−1^, the initial capacitance of the KMO electrode was 216 mAh g^−1^, but it increased to 320 mAh g^−1^ by the tenth cycle (Figure 8a). This effect may be due to the initial activation of the highly crystalline KMO. After 50 cycles, the KMO capacity will be 278 mAh g^−1^, and the MnO_2_ capacity will be 136 mA h g^−1^ (only 60% of the initial capacity) (Figure 8b).

The Rs of KMO cathodes does not change particularly during cycling, but it significantly increases for α-MnO_2_ cathodes, firmly fused with the capacity decrease. Islam et al. also improved the electrochemical performance of MnO_2_ by carbon coating [27]. Other than manganese-based cathodes, vanadium oxide (V_2_O_5_) has been studied extensively as a cathode material for lithium-, sodium-, and zinc-ion batteries. The researchers found some issues such as poor ionic conductivity, low capacity, and structural instability of the V_2_O_5_. Several attempts have been made to solve these problems. Yan et al. [70] improved Li insertion capability into V_2_O_5_ by adding Na ions. It is not only effective for lithium-ion batteries, but this technique is also applicable for other metal-ion batteries. The addition of metal ions (Na^+^, K^+^, Li^+^) act as pillars and enhance the structural stability of V_2_O_5_ in AZIBs [46,47,71,72,73]. Though vanadium-based cathodes offer high capacity, they fail to reach high voltage. In this situation, hybrid ion batteries can reach high potential [74].

## 3. Flexible Zinc-Ion Battery

Zinc has been recognized as one of the most promising anode materials because of its high theoretical capacity, large abundance, low cost, high corrosion resistance in aqueous electrolytes, non-toxicity, low oxidation/reduction potential, environmental friendliness, and good reversibility. However, aqueous zinc-ion batteries (AZIBs) confront some unexpected issues, including water-mediated parasitic reactions that speed up zinc dendrite formation, the dissolution of cathode materials, and the construction of byproducts on the cathode. Hence, these issues arise from the parasitic response, hampering an aqueous zinc battery’s capacity and cyclic stability. Scientists around the globe are working on resolving these issues. They have employed several strategic solutions, such as changing electrolytes, surface engineering, and structural/hierarchical design. Moreover, electrical vehicles and portable devices have been transformed by smart energy storage. The rate of penetration into flexible electronic markets by the present smart energy storage technologies is remarkable. Largely flexible devices require additional requirements, such as bendable, twistable, stretchy, and ultrathin batteries, to adjust mechanical deformation under working conditions. Flexible batteries are essential power sources for these devices. Currently, flexible zinc-ion batteries have attracted more attention for their low cost and considerable energy density [75,76,77,78].

Polymer/hydrogel electrolytes such as poly(vinyl alcohol) (PVA) and polyacrylamide (PAM) can play a significant role in resolving these issues because of the lower content of water in polymer/hydrogel electrolytes. In addition, solid–solid contact between electrodes and the electrolyte rather than a solid–liquid interface can create a significant barrier for preventing the formation of dendrites and the dissolution of cathode materials. Meanwhile, the stable solid–solid interface has unexpected capabilities such as self-healing, elasticity, anti-freezing, and thermal sensitivity [69,75,76,79,80].

Although polymer/hydrogel electrolytes in flexible zinc-ion batteries have achieved tremendous advancements in battery technology, they have some drawbacks, such as ionic activities, length of time effects of polymer/hydrogel electrolytes, ubiquitous charge-transfer mechanisms at the solid–solid interface are unclear, etc. Understanding the mechanisms of polymer gel electrolytes in electrochemical performance is still vague [69,75,76,79,80]. In this section, we will provide recent updates on flexible zinc-ion batteries. To design flexible zinc-ion batteries, it is required to study the basic components of the system, such as the flexible electrodes (cathode and anode), current collector, electrolyte, and separator. Moreover, some important parameters, such as bending properties (geometry), mechanical stability, and energy density, etc., are considered for practical applications. Comprising all the above issues discussed in this section, recent updates on flexible zinc-ion batteries are summarized in Table 2.

### Polymer/Gel Electrolytes

Polymer electrolytes for ZIBs are classified into solid polymer electrolytes, new hydrogel electrolytes, and hybrid polymer electrolytes. In this context, polymer electrolytes surpass aqueous electrolytes to prevent zinc dendrite development and reduce cathode dissolution. In addition, the polymer electrolytes’ additional functionalities lessen parasitic weight invasion in the separator and enhance mechanical performance. Solid polymer electrolytes have more mechanical strength than hybrid polymer electrolytes, although hydrogel electrolytes have a competitive ionic conductivity compared to aqueous electrolytes. Due to their stable structure, solid polymer electrolytes are expected to limit zinc dendrite formation the most. In contrast, hydrogel electrolytes are projected to have a weaker ability to prevent dendrite formation due to the water content in the hydrogel. Combining these benefits, hybrid polymer electrolytes that offer the optimal balance between these viewpoints are critical for further commercialization. They provide high ionic conductivities and multi-competitive functionalities and mechanical behaviors. A three-dimensional, double-cross-linked gelatin and sodium alginate hydrogel imbibed with ZnSO_4_ aqueous solution was used as an electrolyte membrane for flexible Zn-ion batteries, according to Huang et al. [79]. They discovered that the three-dimensional, double-cross-linked gelatin and sodium alginate hydrogel had better electrochemical behavior, easy fabrication, electrochemical stability, and Zn anode suitability [78,99].

**Figure 9:**
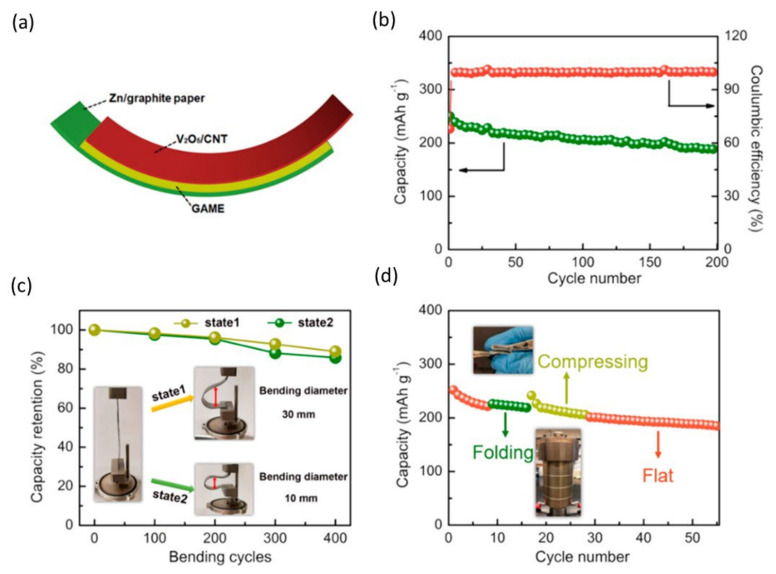
Configuration and performance of a flexible semisolid Zn/V_2_O_5_ battery. (**a**) Schematic diagram of a flexible semisolid Zn/V_2_O_5_ battery. (**b**) Cyclic performance at 2.0 Ag^−1^. (**c**) Capacity retention vs. bending cycle at 2.0 Ag^−1^. The insets show photographs of the battery under two bending states. (**d**) Cyclic performance under various mechanical stimuli at 2.0 Ag^−1^. Reprinted with permission from Ref. [79]. Copyright 2019 American Chemical Society.

**Figure 10:**
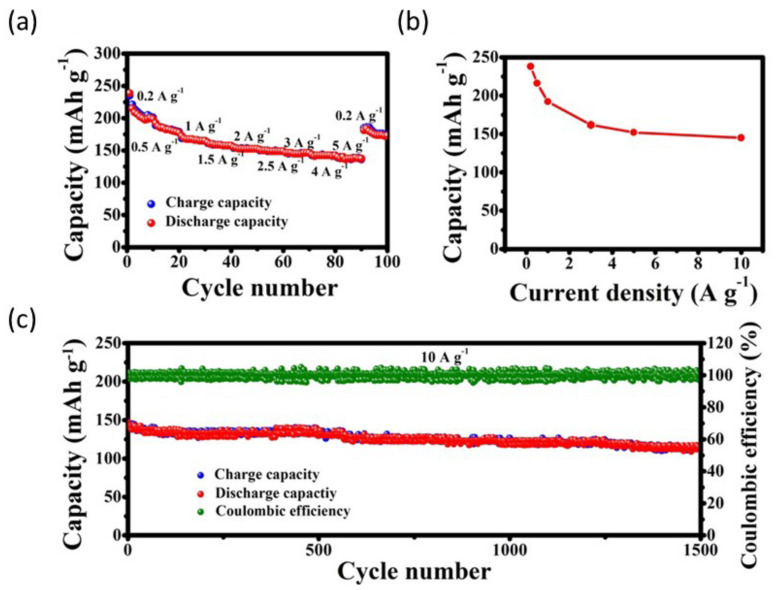
Electrochemical performance of the t-CNTs–PA–PE cathode in ZIBs. (**a**,**b**) Rate performance, and (**c**) cyclic performance at 10 Ag^−1^. Reprinted with permission from Ref. [75]. Copyright 2019 American Chemical Society.

The polymer membrane was well-staged between the V_2_O_5_/CNT cathode and the Zn/graphite paper anode (Figure 9a) [79]. Figure 9b shows the cycle life of a flexible battery at 2.0 Ag^−1^. The battery capacity is 251 mAh g^−1^ after the first cycle and 188 mAh g^−1^ after 200 cycles, with a coulombic efficiency of over 99.8%. Figure 9b,c further show that after 400 bending cycles, the battery can be bent differently with bending diameters of 30 and 10 mm, retaining 89% and 86% of the original capacity, respectively. As shown in Figure 9d, the cell can fold 55 cycles under a load of 1470 times the device’s weight, maintaining 74% of its original capacity. Similarly, poly (3,4-ethylene dioxythiophene) polystyrene sulfonic acid (PEDOT: PSS) is incorporated into polyaniline (PANI) on carbon nanotubes (CNTs) for electrochemical behavior and cycle stability [75]. It shows excellent activity in improving electrochemical behavior. Although the discharge capacity has been shown to gradually decrease with increasing current density, the high conductivity of the tCNTs-PA-PE cathode describes a large capacity of 145 mAhg^−1^ (Figure 10a) even at a sharp current density of 10 Ag^−1^ (Figure 10b) and exemplary cycle stability (Figure 10c), with the reversible capacitance of 113 mAh g^−1^ over 1500 cycles, and remarkable 100% coulombic efficiency (Figure 10d).

**Figure 11:**
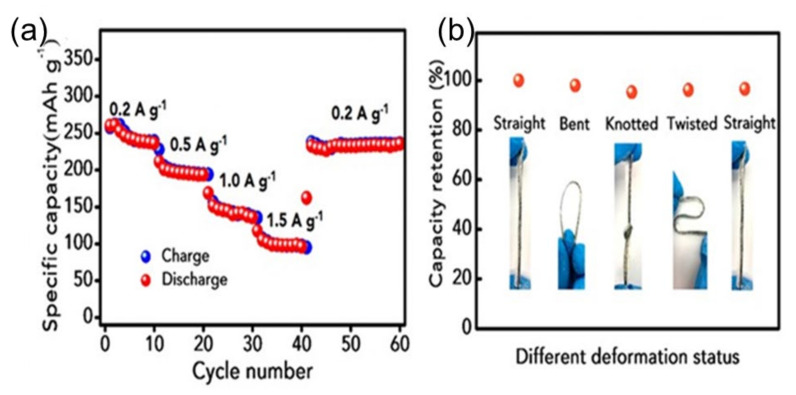
Electrochemical performance of the rechargeable yarn ZIB. (**a**) Rate capabilities at various current densities. (**b**) The capacity retention of the yarn ZIB under various deformation statuses. Reprinted with permission from Ref. [76]. Copyright 2018 American Chemical Society.

The Zn powder carbon film coated on the Zn anode was expected to prevent the abnormal growth of dendrites and promote the performance of Zn. Zhi et al. have shown a stretchable zinc-ion battery (ZIB) with double-helix electrodes and a cross-linked polyacrylamide (PAM) electrolyte [76]. It exhibits a high specific volume, volumetric energy density (302.1 mAh g^−1^ and 53.8 mWhcm^−3^, respectively), and excellent cycle stability (98.5% capacity retention after 500 cycles). In addition, the semisolid-state yarn ZIB showed excellent stretchability (up to 300% elongation) and excellent water tightness (high-capacity retention of 96.5% after 12 h of underwater operation). In Figure 11a, the yarn battery showed high discharge capacities of 260.4, 211.5, 168.7, and 117.7 mAh g^−1^, respectively, at current densities of 0.2, 0.5, 1, and 1.5 Ag^−1^. Most notably, even with a current density of 0.2 Ag^−1^, the average discharge capacity of 235.8 mAh g^−1^ is restored. This corresponds to 96.5% of the initial average capacity (244.3 mAh g^−1^).

Higher efficiency may be due to the consistency of the electrodes and the high ionic conductivity of the PAM-based electrolyte. The deformability test was very sustainable (Figure 11b). The load was evaluated after the trigger. This was almost 97.2% under normal conditions, and it was found that 93.6% of the capacity was stable even after discharge

**Figure 12:**
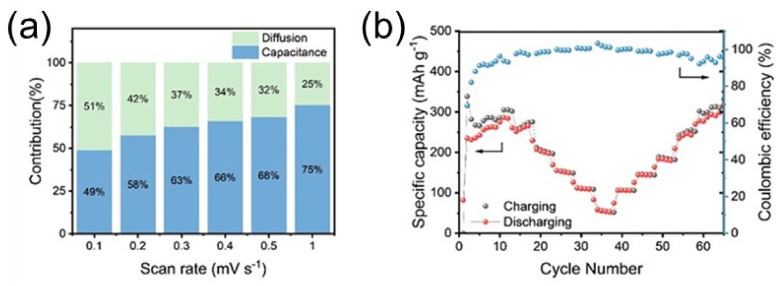
SAPAM electrolyte under the full-cell test: (**a**) diffusion-capacitive control contribution is presented in the bar chart, and (**b**) charge/discharge rate performance of current densities from 0.1 to 5 Ag^−1^. Reprinted with permission from Ref. [80]. Copyright 2021 American Chemical Society.

The hybrid polymer electrolyte was found to have a coulombic efficiency of over 99% and capacity retention of 96% after 1000 cycles at 2 Ag^−1^ according to Parkin et al. [80]. As the sampling rate increased from 0.1 to 1 mVs^−1^, the electrochemical response under capacitive control became much more sensitive, as shown in Figure 12a. As shown in Figure 12b, the charge/discharge rates of SAPAM, SA, and AZIB were studied with gradual current densities ranging from 0.1 to 5 Ag^−1^ over 5 cycles for each current density. As a result, the SAPAM battery achieved an amazing 305 mAh g^−1^ at 0.1 Ag^−1^, and the coulombic efficiency (CE) was 96.2%, while the CE of the original SA battery was 91.6% at 258 mAhg^−1^. In addition, Niederberger et al. used a polydimethylsiloxane (PDMS) substrate and a polyacrylamide (PAM) hydrogel electrolyte, and a capacity of 176.5 mAh g^−1^ was achieved after 120 cycles at various stress levels of up to 50% [100].

**Figure 13:**
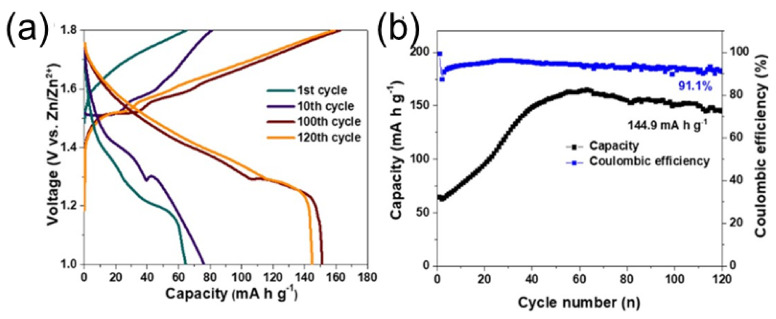
(**a**) Cycling performance of the transparent unstrained battery at a current density of 1 C. (**b**) GCD profiles of the transparent unstrained battery at the 1st, 10th, 100th, and 120th cycles at a current density of 1 C (=308 mA h g^−1^). Reprinted with permission from Ref. [100]. Copyright 2021 American Chemical Society.

**Figure 14:**
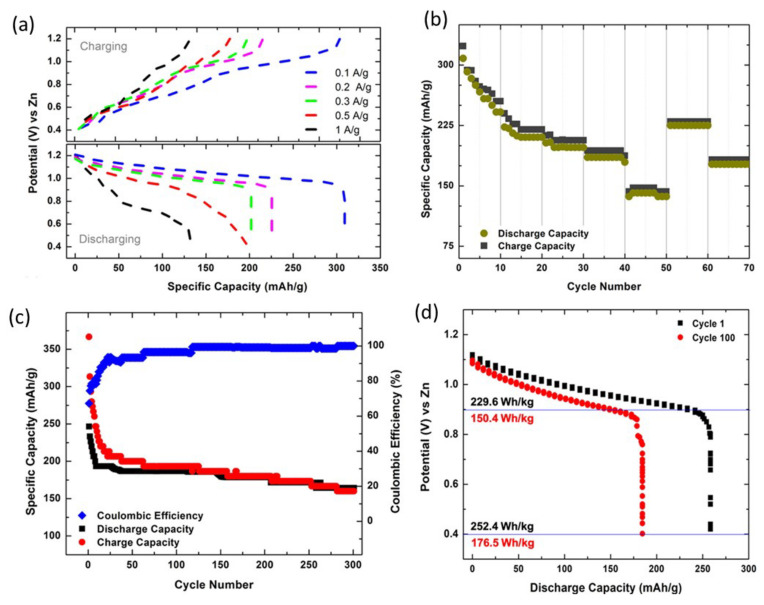
(**a**) Charge–discharge profiles of the assembled cells run at different current densities. (**b**) Rate capability of the Zn-EMD cell with the novel chitosan-alkaline electrolyte cycled between 0.4 and 0.2 V. (**c**) Cycling performance at 0.5 Ag^−1^. (**d**) Discharge curves for cycles 1 and 100 until 0.4 V at 0.5 Ag^−1^ with energy density. Reprinted with permission from Ref. [101]. Copyright 2021 American Chemical Society.

Figure 13a shows the GCD profile obtained at 1 C (= 308 mA g^−1^). In the 1st, 10th, 100th, and 120th cycles, the transparent zinc-ion battery becomes 64.5, 74.4, 151.2, and 144.9 mAhg^−1^. At a current density of 1 C, Figure 13b shows the no-load cycle performance of the transparent cell. According to Madan et al., Figure 12a shows the cycle life of a cell determined by a low-voltage GCD test with a current density of 0.5 Ag^−1^ over 300 cycles [101]. A maximum average specific volume of 248.5 mAh g^−1^ was reported, after which the capacitance value sharply dropped. However, the capacity loss rate after 60 cycles was relatively small, and the average capacity after 300 cycles reached 175 mAhg^−1^. The average capacity has reached 175 mAh g^−1^. The achieved 96.5% coulombic efficiency is also shown in Figure 14a. There was a dramatic decline in the first 15 cycles alone, steadily fluctuating. In addition, Figure 14b shows the discharge curves for cycles 1 and 100 at 0.5 Ag^−1^. Figure 14c shows that the discharge curves at different bending radii align with the original flat state. This shows that the cell works well even when deformed, with a minor loss of capacity.

**Figure 15:**
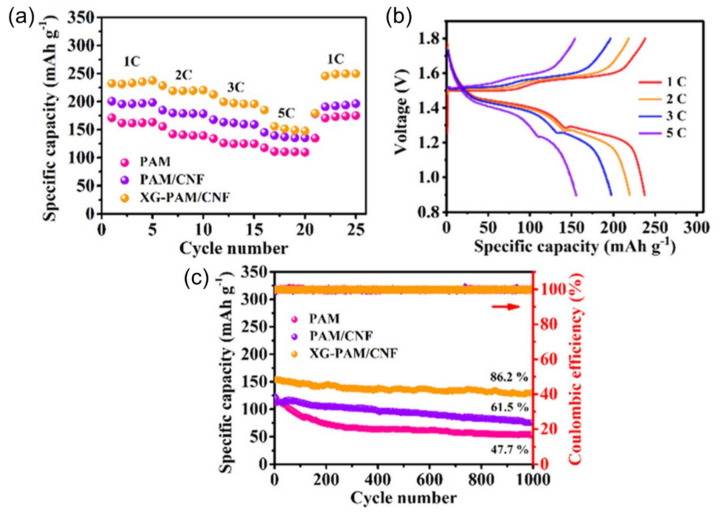
(**a**) Rate capacity of the flexible ZIBs at different rates and (**b**) corresponding charge–discharge curves of the XGPAM/CNF hydrogel. (**c**) Long-term cycle behavior and the corresponding coulombic efficiency of the flexible ZIBs at 4 C. Reprinted with permission from Ref. [102]. Copyright 2020 American Chemical Society.

This shows that the performance stability of the cell is good even under physical bending. In addition, Figure 14d shows the discharge curves for cycles 1 and 100 at 0.5 Ag^−1^. Wang and his group have used the graft copolymer xanthan gum polyacrylamide (XGPAM) and cotton cellulose nanofiber (CNF) combined [102]. They have high ionic conductivity (28.8 mS cm^−1^), better adhesion, specific strength, large and strong ion adsorption, solid specific capacity (237 mAh g^−1^), and excellent cycle stability (86.2% capacity registered at 1000 cycles). The XGPAM/CNF-based ZIB has low bulk and resistivity, indicating a strong electrode–electrolyte interface. This speeds up the electrochemical reaction. As shown in Figure 15a, the rated capacity of the flexible ZIB was also evaluated using three different hydrogel electrolytes. At 1, 2, 3, and 5 C rate, the enhanced specific discharge capacities of 237, 220, 195, and 147 mAh g^−1^, respectively exhibits by the XGPAM/CNF-based ZIB were. Figure 15b shows matching charge/discharge graphs at different rates, whose two plateau functions match the two pairs of redox peaks on the CV curve. In addition, the long-cycle stability of the flexible ZIB based on XGPAM/CNF was remarkable, with a capacity retention of 86.2% after 1000 cycles at 4 C and a coulombic accuracy of almost 100% (Figure 15c).

## 4. Anodes for AZIBs and AFZIBs

**Figure 16:**
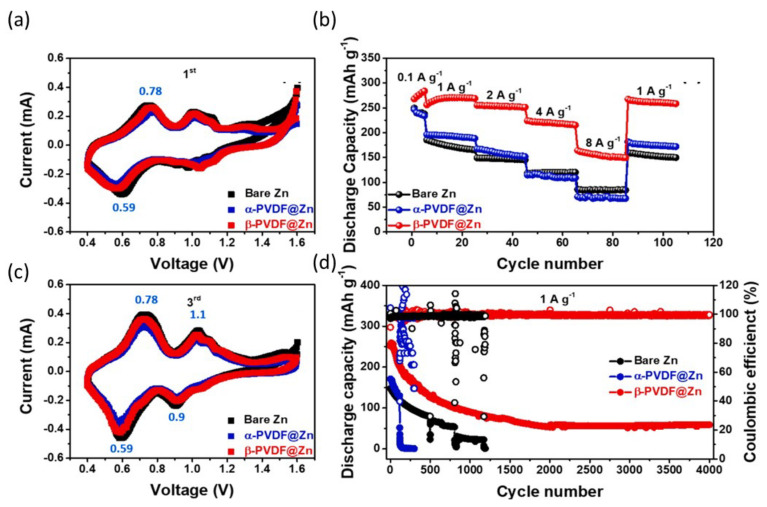
Electrochemical performance of β-PVDF@Zn (red), α-PVDF@Zn (blue), and bare Zn (black) full cells with V_2_O_5_/C cathode: first (**a**) and third (**b**) CV curves at 0.2 mV s^−1^ from 0.4 to 1.6 V. Rate performance (**c**) at various current densities of 0.1, 1, 2, 4, 8, and 1 Ag^−1^, and long-term cycling performance (**d**) at a current density of 1 Ag^−1^. Reprinted with permission from Ref. [103]. Copyright 2021 American Chemical Society.

As previously stated, complications arising from undesired Zn dendrites would lead to prolonged stripping/plating treatments, resulting in water consumption and irreversible negative effects, which could be responsible for degrading the coulombic efficiency (CE) accompanied by an inferior operation life [1,2,3,4,5]. When the Zn dendrite grows at a steady rate, the separator may develop short circuits, posing a security issue. Due to the usage of excessive Zn metal, the discharge rate and consumption proportion of Zn metal anodes are usually poor and do not represent the full energy density. Published studies presently suggest that Zn dendrites may be prevented by modifying the surface of the Zn anode, electrodepositing Zn on 3D nanostructures, adjusting the separator composition, and changing the electrolyte [6,7,8,9,10]. Although such solutions can generally help stabilize cells, the problem of establishing Zn dendrites remains unsolved. Nonetheless, these anode materials have low capacitance, have irreversible phase shifts, and their capacitance drops rapidly during Zn^2+^ insertion/extraction, which severely limits their use. As a result, the creation of an improved insertion anode material for ZIB with a strong structure, high capacitance, and higher power density was very attractive. Hur et al. has used a simple spin-coating process to protect the Zn anode using a protective thin layer of highly polar poly (vinylidene difluoride) (β-PVDF) [103]. The β-PVDF layer is robust and has adjusted the Zn stripping/plating process while withstanding erosion. In the asymmetric cell test, the resulting β-PVDF-coated Zn anode (β-PVDF@Zn) outperformed both bare Zn metal and α-PVDF@Zn at a low overvoltage of 40 mV at 2000 h of operation. In addition, PVDF has extended the life of the entire cell to 4000 cycles while maintaining excellent cycle stability. Two redox peaks of 0.59 and 0.78 were clearly visible in the first cycle (Figure 16a), after which a slightly dark pair of reduction/oxidation peaks in the range 0.9 to 1.1 diverged in each cell. However, after 3 cycles, all peaks in these profiles were integrated into 0.59/0.78 and 0.89/1.14, respectively, according to the Zn^2+^ two-step intercalation/deintercalation process (Figure 16b). The discharge capacities of the batteries using the β-PVDF@Zn anode were 276, 267, 253, 220, and 154 mAh g^−1^ at a high current density of 0.1, 1, 2, 4, and 8 Ag^−1^, respectively, as shown in Figure 16c.

Even after 20 cycles for each period of growing the current rate, the capacity bounced back to 265 mAh g^−1^ when the current density unexpectedly plummeted to 1 Ag^−1^. The β-PVDF cell outperformed the others in terms of rate and cycling, completely overtaking their uninteresting traits. Finally, at a rate of 1 Ag^−1^, the long-term cycle life was assessed (Figure 16d). A cathode material (MnO_2_) ultrathin layer is first coated upon the surface of the carbon cloth as active sites for in situ nucleation and guided nucleation and growth of metal Zn, according to Shao et al. [104]. Consequently, the CC@MnO_2_-UTF@Zn anode can be cycled in an aqueous electrolyte and keep its cycling performance consistent during repeated Zn deposition/stripping procedures. After 100 cycles, the complete cell with the Zn plate anode shows a more extensive capacity loss, with 157 mAh g^−1^ (41.8% capacity retention). Long-term cycling performance was also assessed at 1 Ag^−1^. The complete cell with the CC@MnO_2_-UTF@Zn anode still has a high coulombic efficiency of almost 100% and a specific capacity of 186 mAh g^−1^ after 300 cycles (81.0% of capacity retention). The creation of freestanding, super versatile, and conductive carbon nanotube (CNT)/paper scaffolds to maintain zinc metal anodes has been described by Wang et al. [104]. The scaffold-stabilized zinc anodes had low polarization strengths, a long cycling life of over 1800 h, and superior charging–discharging capabilities.

**Figure 17:**
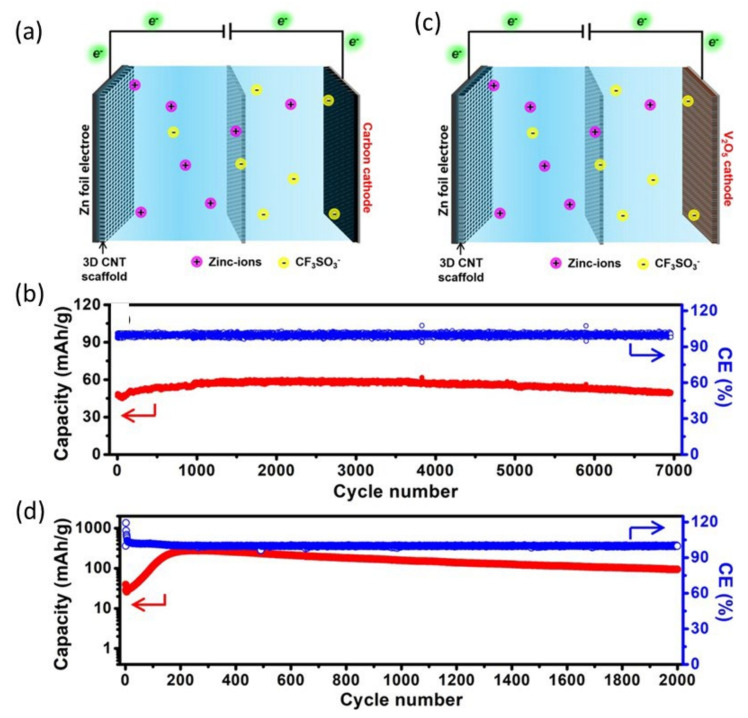
(**a**) Schematics, (**b**) cycling performance of activated carbon|Zn(CF_3_SO_3_)_2_|Zn@CNTS ZICs, (**c**) schematics, and (**d**) cycling performance of Zn@CNTS|Zn(CF_3_SO_3_)_2_|V_2_O_5_ ZIBs. Reprinted with permission from Ref. [105]. Copyright 2020 Elsevier.

Zinc-ion batteries/hybrid capacitors with ultralong cycle lifetimes were also successfully produced, due to the scaffold-stabilized zinc anodes’ robust electrochemical stability and reversibility [105]. The V_2_O_5_|Zn(CF_3_SO_3_)_2_|Zn@CNTS ZICs have been charged/discharged at 3 Ag^−1^ for 2000 cycles and the activated carbon|Zn(CF_3_SO_3_)_2_|Zn@CNTS ZIBs have been charged/discharged at 2 Ag^−1^ for 7000 cycles (Figure 17a–d). Both have an excessive degree of cyclical balance. A unique 2D ultrathin-layered zinc orthovanadate array cathode, a Zn array anode supported with the aid of conductive porous graphene foam, and a gel electrolyte were used by Fan et al. to develop a high-end, ultra-strong, flexible, quasi-solid-country zinc-ion battery [82]. Each electrode has a nanoarray construction that ensures high-fee functionality and prevents dendrite formation.

**Figure 18:**
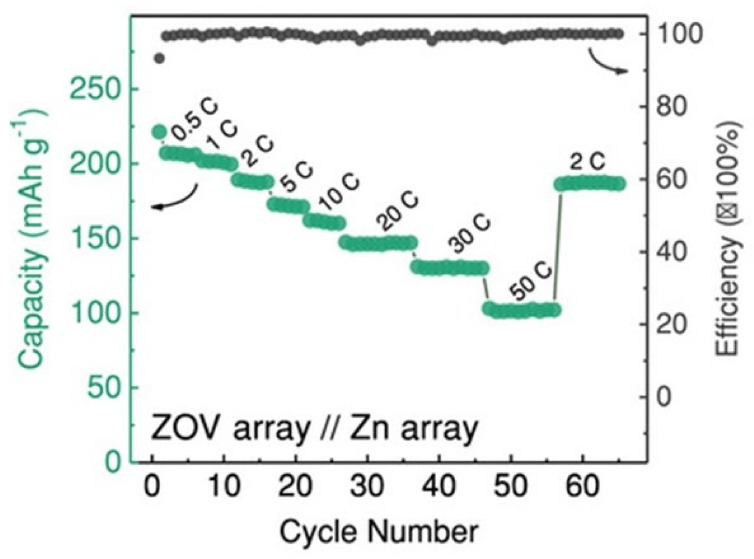
Quasi-solid-state ZIB performance with ZOV array as a cathode and Zn array as an anode. The rate capability of the cell at varied current densities, from 0.5 to 50 C. Reprinted with permission from Ref. [82]. Copyright 2018 Wiley.

The porous, thin, ZOV nanoarray shape with uncovered 2D ion channels is vital in this regard to enable ions to gain entry to and velocity-fee switch at the electrode/electrolyte interface with a short Zn^2+^ and electronic transport direction. Figure 18 demonstrates the excessive price of the overall performance. At 10 C, the release abilities are greater than 160 mAh g^−1^, and 101 mAh g^−1^ at 50 C. Subsequently, even at high fees, our QSS-ZIB demonstrates correct cycling sustainability. After 800 cycles, the capability stays flat, and after 2000 cycles (89% of the preliminary value), a selected capability of 125 mAh g^−1^ may nevertheless be maintained at 20 C.

From the above discussion, it can be concluded that similar types anode can be used for both AZIBs and AFZIBs. However, special designated flexible anodes are required for AFZIBs, whereas Zn foil can be used for AZIBs. No supporting current collector is needed while using Zn foil as an anode. In contrast, Zn-containing carbonaceous compounds such as Zn@Graphene, Zn@CNT, etc., are directly used as anode materials, and carbon materials can act as current collectors. This kind of anode is more effective in AFZIBs.

## 5. Challenges of Flexible Zinc-Ion Battery

**Figure 19:**
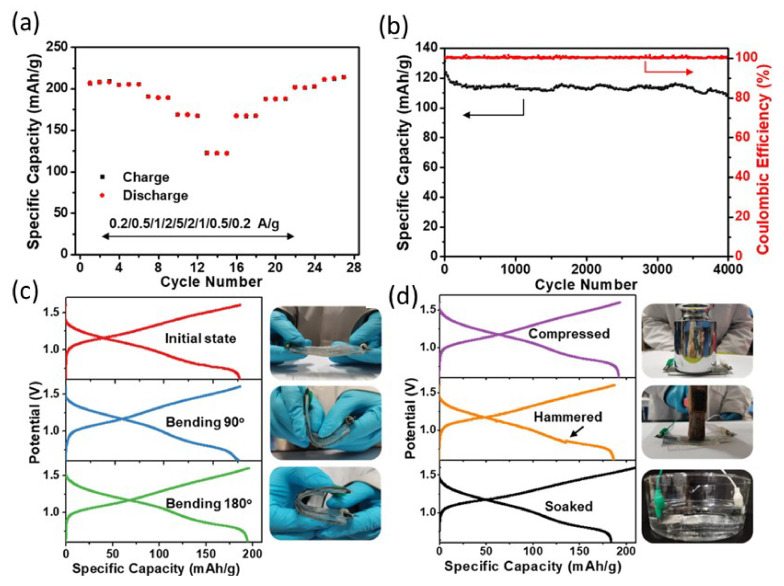
Electrochemical performance of OHE-based Zn||PANI battery at room temperature. (**a**) Cycling performance at various current densities. (**b**) Long-term cycling performance at 5.0 Ag^−1^ for 4000 cycles. (**c**,**d**) Charging and discharging stability of the OHE-based battery under a series of harsh working conditions. Reprinted with permission from Ref. [106]. Copyright 2021 American Chemical Society.

Due to its outstanding performance and stretching/bending characteristics, the flexible zinc-ion battery is a promising invention for future commercial battery technology [37]. However, appropriate electrolytes and suitable compatible electrodes are the critical issues for the sustainable development of FIBs. Although extensive research has been carried out in these areas, it is still in its infancy. Therefore, we have discussed some recent updates about electrolytes and electrodes for AFZIBs. Electrolytic engineering is a promising way to make sustainable FIBs. Even though flexible zinc-based batteries work well in the lab, they are unable to find real-world applications, partly due to the substandard performance of the hydrogel electrolytes under dire circumstances. For example, hydrogels, which have high ionic conductivity and good flexibility, are one of the most promising flexible electrolytes for flexible zinc-based batteries. Their innate water-saturated polymeric networks facilitate diffusion of ions, resulting in greater ionic conductivity, hydrogel interfaces ensure strong interaction with electrodes, and their dimensionally stable properties enable flexible zinc-based batteries to be used in a wide range of applications. Nevertheless, the ramifications of harsh operational conditions on the battery’s quality and longevity, which are crucial for implementing flexible zinc-based batteries, are still to be studied. Furthermore, the hydrogel electrolyte is more sensitive to the environment than electrodes in the flexible zinc-based battery system due to its fragile nature and water-saturated body. For loose zinc-based batteries, the revocation of hydrogel electrolytes at severe temperature changes and diverse anomalies are still a concern. Liu et al., for instance, discovered that at ambient temperature, organohydrogel electrolytes (OHEs) have superior performance (122.1 mAh g^−1^, 5 Ag^−1^) and sturdy cycle stability (81.5%, 4000 cycles) [82]. This is attributed to the high-performance change examined from 0.2 to 5 Ag^−1^ via editing the contemporary density (Figure 19a). The primarily OHE-based Zn||PANI battery has desirable excessive-fee-cycling overall performance, as may be validated [106]. The specific capacity of the OHE-based total battery is 207.7 mA h g^−1^ at a 0.2 Ag^−1^ contemporary density. After 4000 cycles at a high cutting-edge density of 5 Ag^−1^, the particular capability of the OHE-based Zn||PANI battery remains at 108.4 mA h g^−1^, displaying a potential retention charge of more than 80%. In the cycle technique, the coulombic performance is almost 100% (Figure 19b), confirming that the primarily OHE-based Zn||PANI battery is electrochemically solid within the voltage range of 0.6–1.6 V, and that no unfavorable reactions take place. The security of OHE-based batteries as flexible strength storage systems was then tested by subjecting the OHE-based Zn||PANI batteries to rigorous operational situations. The primarily OHE-based battery keeps a consistent price and discharge conduct, as illustrated in Figure 19c,d, and a similar charge–discharge profile, observed even in adverse situations. Although the cell is bent at different angles (90°, 180°, etc.) and is placed in different conditions (compressed, hammered, soaked), it can deliver similar capacity at 90° and 180° bending angles.

**Figure 20:**
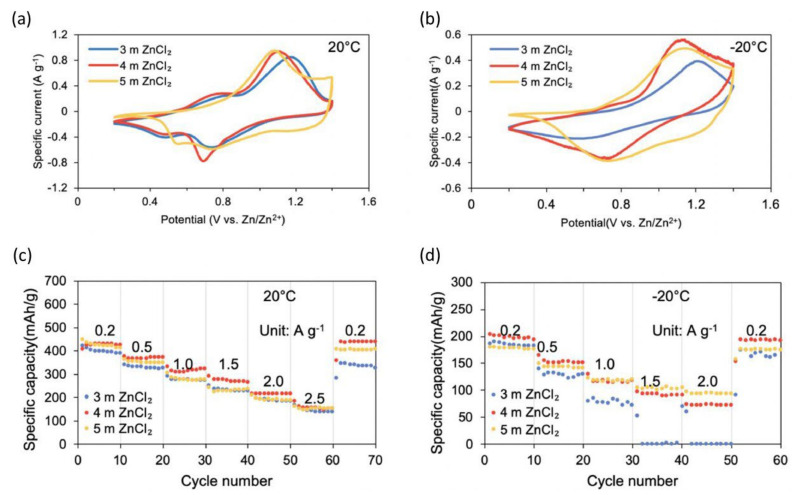
Under 0.5 mV s^−1^, the CV curves of the batteries based on the hydrogel electrolytes prepared using 3/4/5 m ZnCl_2_ combined with 1.2 g of xanthan gum at (**a**) 20 °C and (**b**) −20 °C. Rate performances of the batteries based on the electrolytes prepared using 1.2 g of xanthan gum combined with 3/4/5 m ZnCl_2_ solution at (**c**) 20 °C and (**d**) −20 °C. Reprinted with permission from Ref. [107]. Copyright 2021 Elsevier.

However, within the flexible zinc-based battery system, the hydrogel electrolyte seems more environmentally sensitive than the electrode fracture toughness and water-saturated body. Hence, two things occur if the temperature goes below 20 °C, and then the ionic conductivity of the hydrogel electrolyte dramatically reduces. Dehydration occurs if the temperature goes above 50 °C. Structure deformation, salt crystallization, and decreased ionic conductivity result from the simple dehydration of the hydrogel at extremely high temperatures (50 °C). Chen et al. have shown a flexible zinc-ion battery consisting of an optimized concentrated hydrogel electrolyte sandwiched between an ultrathin zinc anode and an NH_4_V_3_O_8_·1.9H_2_O cathode for enhanced performance at a subzero temperature [107]. The resultant flexible battery performs perfectly when a concentrated hydrogel electrolyte is synthesized using 1.2 g of xanthan gum saturated in 4 m of ZnCl_2_ solution, having an extremely high capacity of 201 and 83 mAh g^−1^ under 0.2 Ag^−1^ at −20 and −40 °C, respectively. Figure 20 illustrates the electrochemical behavior of batteries relying on these condensed hydrogel electrolytes at 20 and −20 °C. Under 0.5 mVs^−1^ at 20 °C, Figure 20a shows the CV curves of the batteries incorporating hydrogel electrolytes synthesized with 1.2 g of gum and 3, 4, or 5 m of salt solution, respectively, while Figure 20b shows the CV curves of the two batteries. The battery with the hydrogel electrolyte prepared with 4 m of salt solution generates a CV curve with wider regions and sharper peaks. The proportion of zinc ions intercalated into the electrode measures the scale of the CV curve, with a greater area showing more significant zinc ions intercalated into the electrode. The width of peaks is determined by the restricting phase of mechanisms (mass or electron transfer velocity); consequently, sharper peaks indicate faster transport kinetics during this scenario. At −20 °C, the variation between the CV curves of the two batteries is even greater, indicating better electrochemical performance and quicker and more efficient transport kinetics from the 4 m hydrogel electrolyte, especially.

**Figure 21:**
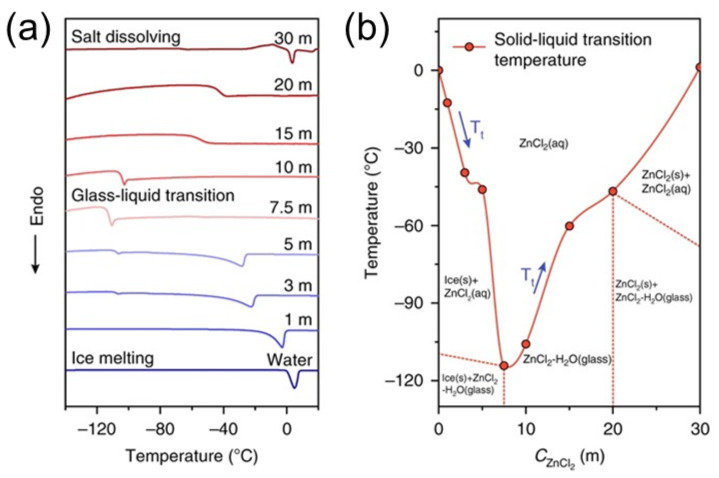
The properties of solid–liquid transitions and the mechanism of vitrification. Thermodynamic change and transition of different C_ZnCl2_ electrolytes at low temperatures. (**a**) DSC test from −150 to 20 °C at a heating rate of 5 °C min^−1^. (**b**) The major Tt vs. C_ZnCl2_ and the phase composition of ZnCl_2_ solution at different temperatures and concentrations. Reprinted with permission from Ref. [108]. Copyright 2020 Nature.

At −20 °C, the variation between the CV curves of the two batteries is even greater, indicating better electrochemical performance and quicker and more efficient transport kinetics from the 4 m hydrogel electrolyte, especially at subzero temperatures, plausibly due to greater ionic conductivity due to the elevated salt concentration. The operations of the batteries incorporating hydrogel electrolytes made with 1.2 g of gum and 3, 4, or 5 m of salt solution, correspondingly, at 20 °C are shown in Figure 20c, whereas the c-rate performance of these three batteries at −20 °C are shown in Figure 20d. Chen et al. reported breaking the initial hydrogen-bond network in ZnCl_2_ solution by altering the electrolyte structure, inhibiting water freezing, and lowering the aqueous electrolyte’s solid–liquid temperature within the range from 0 to −114 °C [68]. This ZnCl_2_-based low-temperature electrolyte enables polyaniline||Zn batteries to function in an exceeding temperature range of −90 to 60 °C, which covers the complete planet’s surface temperature range. These polyaniline||Zn batteries are durable at −70 °C (84.9 mA h g^−1^) and have capacity retention of 100% after 2000 cycles. The ionic conductivities of the several C_ZnCl2_ electrolytes (1, 5, 7.5, 10, and 30 m) and also the standard electrolyte (2 m ZnSO_4_ and a few m Zn(CF_3_SO_3_)_2_ electrolyte) were investigated within the temperature range of −100–60 °C to enhance and choose the optimum LTE with optimum ionic conduction at cold temperatures (Figure 21a). Regardless of the fact that the 1 and 5 m ZnCl_2_ electrolytes block at −12.6 and −46.0 °C, correspondingly, they need a robust ionic conductivity at cold temperatures, due to ionic conduction facilitated by the concentrated electrolyte below their major Tt. Conversely, solid ice obstructs ionic conduction and reduces ionic conductivities by a tenth compared to the frozen-solid 7.5 m ZnCl_2_ electrolyte at −80 °C. Although the T_t_ of the 10 m ZnCl_2_ electrolyte is low below −100 °C, it also features a quicker ionic conductivity reduction than the 7.5 m ZnCl_2_ electrolyte. The 7.5 m ZnCl_2_ electrolyte showed a high ionic conductivity of 1.79 mS cm^−1^ at −60 °C and 0.02 mS cm^−1^ at −100 °C thanks to the extended liquid-phase temperature range and relatively low concentration. The activation energies of ionic conduction in electrolytes were computed using the Arrhenius equation to quantitatively display the change of ionic conductivity with temperature. As illustrated in Figure 21b, there are two stages, one at high temperatures and the other at cold temperatures, both with various activation energies.

The mechanical stability of the electrode during lengthy displacement and the unexpected dendrite expansion during cycling are the most important issues encountered by zinc metal anodes, which tend to end in poor cycle performance and coulombic efficiency of the battery, restricting the lifetime of flexible zinc-ion batteries. Zinc may be a better electrode material than lithium because it is less costly, features a high percentage within the crust, and features a smaller redox equilibrium potential. Furthermore, because zinc is not sensitive to the environment, it is cheaper to fabricate and package zinc-based batteries than lithium-based batteries. Traditional zinc-based batteries, such as Zn-MnO_2_, Zn-Ni, and Zn-Air, currently exist on the market, however, they are mostly hard and applied in non-flexible electronic devices. Several studies have been conducted to make these batteries more adjustable. Furthermore, ZIBs have a bright future in wearable electronics thanks to their increased energy density, relatively low costs, environmentally friendly nature, reliability, and other advantages. ZIBs have received a lot of interest as a possible alternative to lithium-ion batteries. Besides that, the difficulties encountered by zinc metal anodes are the electrode’s reliability value during lengthy displacement and unrestrained dendrite growth throughout cycling, leading to poor cycle effectiveness and battery coulombic efficiency, limiting the expected lifespan of flexible zinc-ion batteries, and negatively impacting their useful application.

## 6. Conclusions

Cathodes’ dissolution and dendrite formation from aqueous electrolytes in an aqueous zinc-ion battery can negatively affect the storage capacity and cyclic stability. However, several approaches have been applied to form sustainable AZIBs, but they are still in their infancy. A flexible zinc-based battery is a promising technology because of gel electrolytes. Since they behave as electrolytic conductors and electrode separators, gel electrolytes are an important part of the flexible zinc-based battery. The influences of severe working conditions (i.e., cold and hot temperatures, physical deflections) on gel electrolytes and zinc-based batteries were systematically reviewed in this paper. Likewise, we introduced possible approaches and summarized their existing issues. It is proving to be useful to ascertain the battery function in quite a broad temperature range for an extended length of time without having to sacrifice other electrochemical behavior, which is completely essential for real implementation.

## Figures and Tables

**Table 1:** The recent updates of Mn-based cathode materials.

Cathode Materials	Electrolyte	Cyclability (mAh g^−1^)	Ref.
α-MnO_2_	1 M ZnSO_4_	100 after 100 cycles at 6C (*n*C = a full discharge in 1/*n* h)	[34]
α-MnO_2_	1 M ZnSO_4_	140 after 30 cycles at 10.5 mA g^−1^	[58]
α-MnO_2_–CNT	2 M ZnSO_4_	100 after 500 cycles at 5000 mA g^−1^	[61]
α-MnO_2_	1 M ZnSO_4_	130 after 30 cycles at 42 mA g^−1^	[62]
α-MnO_2_	1 M ZnSO_4_	147 after 50 cycles at 83 mA g^−1^	[29]
γ-MnO_2_	1 M ZnSO_4_	158 after 40 cycles at 0.5 mA cm^−2^	[11]
α-MnO_2_	1 M ZnSO_4_	104 after 75 cycles at 83 mA g^−1^	[26]
V-doped α-MnO_2_	1 M ZnSO_4_	131 after 100 cycles at 66 mA g^−1^	[63]
α-MnO_2_@C	1 M ZnSO_4_	189 after 50 cycles at 66 mA g^−1^	[27]
β-MnO_2_	1 M ZnSO_4_	135 after 200 cycles at 200 mA g^−1^	[12]
β-MnO_2_	3 M	135 after 2000 cycles at 6.5C (*n*C = a full discharge of 308 mA g^−1^ in 1/n h)	[64]
PANI-δ-MnO_2_	2 M ZnSO_4_	280 after 200 cycles at 200 mA g^−1^	[65]
MnO_2_	1 M ZnSO_4_	1.67 mAh cm^−2^ after 1800 cycles at 60 mA cm^−2^	[66]
δ-MnO_2_	1 M ZnSO_4_	175 after 1000 cycles at 3096 mA g^−1^	[59]
Mn-deficient ZnMn_2_O_4_	2 M ZnSO_4_ + 0.2 M MnSO_4_	100% capacity retention after 1000 cycles at 3 A g^−1^	[67]

**Table 2:** The recent updates of the flexible zinc-ion battery.

Cathode Materials	Electrolyte	Capacity	Energy Density	Cycling Stability	Ref.
Co(III) rich-Co_3_O_4_@CC	ZnSO_4_/CoSO_4_/ PAM gel	52 (8 A ^g−1^)	360.8 Wh kg^−1^ (0.5 A g^−1^)	94.6% capacity retention 2000 cycles (2 A g^−1^)	[81]
ZOV@graphene foam	ZnSO_4_/fumed Silica	204 (0.5 C)	115 Wh kg^−1^	89% capacity retention 2000 cycles (20 C)	[82]
MnO_2_@PEDOT@CC	ZnCl_2_/MnSO_4_/ PVA gel	282.4 (0.7 A g^−1^)	504.9 Wh kg^−1^	77.7% capacity retention after 300 cycles	[83]
Expanded MoS_2_@CC	ZnSO_4_/starch-g- PAM gel	202.6 (0.1 A g^−1^)	148.2 Wh kg^−1^	98.6% capacity retention after 600 cycles	[84]
NaV_3_O_8·_1.5H_2_O@the steel meshes	Na_2_SO_4_/ZnSO_4_/ gelatin gel	160 (0.5 A g^−1^)	144 Wh kg^−1^	77% capacity retention after 100 cycles (0.5 A g^−1^)	[85]
MnO_2_@the CNT paper	ZnSO_4_/MnSO_4_/ gelatin-g-PAM gel	306	6.18 mW h cm^−2^	97% capacity retention after 1000 cycles (2772 mA g^−1^)	[86]
MnO_2_@CC	ZnSO_4_/MnSO_4_/ gelatin gel	265 (1 C)	-	76.9% capacity retention after 1000 cycles	[87]
MnO_2_@nitrogen-doped CC	ZnCl_2_/MnSO_4_/ PVA gel	353 (0.5 A g^−1^)	440 Wh kg^−1^	86.7% capacity retention after 1000 cycles	[7]
MnO_2_/rGO@CC	ZnSO_4_/MnSO_4_	332.2 (0.3 A g_−1_)	456.2 Wh kg^−1^	96% capacity retention after 500 cycles (6 A g^−1^)	[88]
Freestanding graphene/ VO_2_ composite films	Zn(CF_3_SO_3_)_2_	194 (8 A g^−1^)	65 Wh kg^−1^	99% capacity retention after 1000 cycles	[89]
MnO_2_@CNT film	ZnSO_4_/MnSO_4_/ xanthan gel	260 (1 C)	364 Wh kg^−1^	90% capacity retention after 330 cycles (1 C)	[90]
Polyaniline@carbon Felts	Zn(CF_3_SO_3_)_2_/ PVA gel	109 (0.5 A g^−1^)	-	91.7% capacity retentions after 200 cycles	[91]
MnO_2_@CNT fiber	Zn(CF_3_SO_3_)_2_/ PVA gel	290 (0.1 A g^−1^)	360 Wh kg^−1^	75% capacity retention after 300 cycles	[92]
MnO_2_@PPy@ stainless Steel	ZnSO_4_/MnSO_4_/ gelatin–borax gel	135.2 (1 C)	-	87% capacity retention after 500 cycles, 60% capacity retention after 1000 cycles	[93]
MnO_2_@CNT yarn	ZnSO_4_/MnSO_4_/ PAM gel	302.1	53.8 mWh cm^−3^	98.5% capacity retention after 500 cycles	[94]
ZnHCF@MnO_2_@Ni foil	ZnSO_4_/PVA gel	89 (100 mA g^−1^)	149 Wh kg^−1^	71% capacity retention after 500 cycles (400 mA g^−1^)	[95]
Husk-like α-MnO_2_	1 M ZnSO_4_ + 0.1 M MnSO_4_ Gel	321 (0.33mAg^−1^)		100 mAh g^−1^ after 100 cycles (333 mA g^−1^)	[96]
Ce-MnO_2_@CC	polyacrylamide–ZnSO_4_–MnSO_4_(PAM/ZnSO_4_-MnSO_4_) gel	311 mAh g^−1^ at (100 mA g^−1^)	370 Wh kg^−1^	106 mAh g^−1^ (2000 mA g^−1^)	[97]
Nanocellulose-Based Hybrid Hydrogels	3 M ZnCI/2M NH_4_CI/1 M ZnSO_4_ Solid electrolyte	149.4 mAh/g (0.5 A/g)	113.2 mWh/g	96% capacity retention after 1000 cycles	[98]
